# COVID-19 vaccines: progress and understanding on quality control and evaluation

**DOI:** 10.1038/s41392-021-00621-4

**Published:** 2021-05-18

**Authors:** Qunying Mao, Miao Xu, Qian He, Changgui Li, Shufang Meng, Yiping Wang, Bopei Cui, Zhenglun Liang, Junzhi Wang

**Affiliations:** National Institutes for Food and Drug Control, Beijing, China

**Keywords:** Drug regulation, Biologics

## Abstract

The outbreak of COVID-19 has posed a huge threat to global health and economy. Countermeasures have revolutionized norms for working, socializing, learning, and travel. Importantly, vaccines have been considered as most effective tools to combat with COVID-19. As of the beginning of 2021, >200 COVID-19 vaccine candidates, covering nearly all existing technologies and platforms, are being research and development (R&D) by multiple manufacturers worldwide. This has posed a huge obstacle to the quality control and evaluation of those candidate vaccines, especially in China, where five vaccine platforms are deployed in parallel. To accelerate the R&D progress of COVID-19 vaccines, the guidances on R&D of COVID-19 vaccine have been issued by National Regulatory Authorities or organizations worldwide. The Center for Drug Evaluation and national quality control laboratory in China have played a leading role in launching the research on quality control and evaluation in collaboration with relevant laboratories involved in the vaccine R&D, which greatly supported the progression of vaccines R&D, and accelerated the approval for emergency use and conditional marketing of currently vaccine candidates. In this paper, the progress and experience gained in quality control and evaluation of COVID-19 vaccines developed in China are summarized, which might provide references for the R&D of current and next generation of COVID-19 vaccines worldwide.

## Introduction

COVID-19 is an infectious disease with arguably the greatest impact on human society and economy in the past century. Up to now, the number of COVID-19 cases recorded have exceeded 100 million worldwide and the death toll has surpassed 2 million.^1^ Vaccination is the most effective means to control the COVID-19 epidemic. Consequently, >200 companies and institutions quickly put together plans for the research and development (R&D) of COVID-19 vaccines, covering almost all technology platforms available for preparing vaccines.^2,3^ Currently, COVID-19 vaccines from 19 developers have entered phase III clinical trials, and 12 related vaccines have been approved for conditional marketing or emergency use worldwide (Table 1).^4–33^ Matters pertaining to the quality control and evaluation of the efficacies and safety of these new vaccines emanating from different platforms pose numerous challenges. In particular, there is very little knowledge and information on the type of parameters that should be included in the quality control and evaluation of vaccines like mRNA vaccines produced, using never before used technology routes. Even for relatively mature technology platforms like inactivated vaccines and recombinant protein-based vaccines, the specifications for the vaccine and methods used by different enterprises are difficult to standardize and unify for comparison. This could be partly attributed to the urgent need and poor knowledge on basis research for rolling out a vaccine, leading to a rushed research, evaluation, and the development of quality control system. How to timely update and improve the relevant specifications, and guidelines pertaining to quality control and evaluation of vaccines as new data from R&D and in-field application becomes available remains a challenge. In addition, establishment of the normative requirements for the second generation and COVID-19 vaccines effective against mutants are other pressing issues currently facing regulatory authorities.

**Table 1:** Progress of representative COVID-19 vaccines developed by different platforms

Vaccine types	Advantages and disadvantages	Representative	R&D institutions	R&D progress
Inactivated virus vaccine	Well-established R&D pipeline, easy and fast to prepare; needs multiple inoculations to strengthen, not easy to stimulate T-cell immunity	BBIBP-CorV^4^	Sinopharm (Beijing)	On market^8^
		CoronaVac^5^	SinoVac	On market^9^
		COVILO^6^	Sinopharm (Wuhan)	On market^9^
		BBV152^7^	Bharat Biotech	Clinical III^10^
Nucleic acid vaccine (DNA or RNA)	R&D platforms are generalizable and easily mass produced; DNA route may have potential genetic safety risks; mRNA is unstable; no precedent for either marketed vaccine	mRNA-1273^11,12^	Moderna	On market/EUA^16–18^
		BNT162b2^13,14^	BioNTech	On market/EUA^19–21^
		CVnCoV INO-4800^15^	Curevac AG	Clinical II/III^10^
			Inovio	Clinical II/III^10^
Viral vector vaccine	Can induce strong humoral and cellular immunity; Interferes with the preexisting immune response to vector viruses	Ad5-nCoV^22,23^	CanSino	On market^9^
		ChAdOx1-nCoV-19^24–26^	AstraZeneca	On market/EUA^29^
		Sputnik V^27^	Gamaleya	On market^30^
		Ad26.COV2.S^28^	Janssen Pharm	EUA^31^
Recombinant protein vaccine	Clear composition, high safety and stability; Weak immunogenicity and adjuvants are needed	NVX-CoV2373^32^	Novavax	Clinical III
		ZF2001^33^	Zhifei Biological	Clinical III/EUA^10^
Live attenuated vaccine	Long duration of immune maintenance with good results; Long development cycle, variable criteria, possible mutations	COVI-VAC	Codagenix/Serum Institute of India	Clinical I^10^

In China, five kinds of vaccine platforms are being explored in parallel. There are 17 vaccines at various stages of clinical trials. Among these, six vaccines have entered phase III clinical trials. At the time of the writing of this manuscript, four vaccines have been approved for the marketing or emergency use in China and many other countries around the world. Regulations for the quality control and evaluation of COVID-19 vaccines are very stringent in China and were developed painstakingly, while working in close cooperation with different stake holders. Therefore, the achievements and experiences gained in China on COVID-19 vaccines could serve as a guide for the development and application of vaccines worldwide. In this paper, the progress and experience gained while working on the quality control and evaluation aspects of COVID-19 vaccines developed are summarized, especially in China. The considerations involved in establishing a standardized quality control and evaluation system to meet the technical requirements of quality at different stages of R&D are proposed.

## Technical guidelines for quality control and evaluation of COVID-19 vaccines

To guide and standardize the R&D of COVID-19 vaccines, global health organizations or regulatory authorities, including WHO, NMPA of China, FDA of the United States, EMA of the European Union, and PMDA of Japan, have issued a framework for the evaluation of clinical studies and guidelines related to emergency use of COVID-19 vaccines (Table 2).^34–45^ As other vaccines, relevant requirements on chemistry, manufacturing, and controls have been put forward in these guidelines. In terms of quality control, critical source materials (such as cell banks and virus banks), quality control system, analytical methods, and qualification/validation data and validation data for assays used to evaluate critical vaccine qualities, such as purity, identity, and potency for all stages of manufacturing should be clarified and provided.^40^ While promulgating the technical guidelines for the R&D of COVID-19 preventive vaccines, China has formulated the technical guidelines for the pharmaceutical research of COVID-19 preventive mRNA vaccines to address and meet the growing interest in the R&D of new mRNA vaccines.^37^ These technical guidance documents have played an important role in the development and approval of vaccines for emergency responses globally. However, most of the above guidelines are based on the existing experience in vaccine development, which need to be updated and revised in a timely manner based on new data and trends arising from the research on the produce, quality control, preclinical, and clinical trials related to COVID-19 vaccines.

**Table 2:** Technical guidelines for the research and development of COVID-19 vaccines published worldwide

Agency/Organization	Guidance
World Health Organization (WHO)	Relevant WHO technical documents for COVID-19 vaccines and other biologicals^34^
	Considerations for evaluation of COVID-19 vaccines^42^
Food and Drug Administration (FDA)	Development and licensure of vaccines to prevent COVID-19^36^
	Emergency use authorization for vaccines to prevent COVID-19^43^
National Medical Products Administration (NMPA)	Guiding principles for research and development technical of COVID-19 preventive vaccines (trial)^38^
	Guiding principles for pharmaceutical research technical of COVID-19 preventive mRNA vaccines (trial)^37^
	Guiding principles for clinical evaluation of COVID-19 preventive vaccines (trial)^40^
	Guiding principles for clinical study technical of COVID-19 preventive vaccines (trial)^41^
	Technical points for research and evaluation of nonclinical efficacy for COVID-19 preventive vaccines (trial)^39^
European Medicines Agency (EMA)	EMA considerations on COVID-19 vaccine approval^44^
	Quality-related scientific guidelines that EMA considers most relevant for COVID-19 vaccine developers^35^
Pharmaceuticals and Medical Devices Agency (PMDA)	Principles for the evaluation of vaccines against the novel coronavirus SARS-CoV-2^45^

In addition, in view of the emergence of new problems and challenges, it is necessary to formulate contingent technical guidelines that can be tested quickly and adopted for standardization. For example, WHO and FDA are currently developing guidelines on testing the impact of mutant strains on the efficacy of protection of vaccines. Relevant departments in China are also working on preparing supplemental guidelines and policies to assist and streamline responses to rapidly evolving situations during pandemics.

## Vaccine quality standards and evaluation techniques

Countries all over the world had to rush to come up with countermeasures to mitigate the impact of the COVID-19 pandemic. Companies and institutions scrambled for the development of vaccines. The resulting tight R&D time lines, adoption of numerous platforms for the development of vaccines, participation of multiple enterprises, and inadequate scale-up, as well as production facilities severely impacted the setting up and implementation of standards to guide the development of COVID-19 vaccines through the various stages of R&D and production. For instance, in China itself, currently, there are >50 R&D enterprises working on five different vaccine platforms. Among the candidates being studied and developed, 17 products have entered clinical trials. Standardization of the various processes developed by many companies posed a huge challenge to the Chinese regulatory authorities. To address this, the Chinese Center for Drug Evaluation (CDE) has released multiple publications on guiding principles for vaccine development, recommendations on methods, and the statutory standards for the quality control at each step of R&D. Furthermore, the national laboratory of China, National Institutes for Food and Drug Control (NIFDC), worked closely with several laboratories conducting research on vaccines to establish a series of quality control and reference standards for the purposes of evaluation, which provide valuable support for vaccine development.

### Setting up quality control standards and key items

The purpose of recommending guidelines for vaccine quality is primarily to ensure the safety and effectiveness of vaccines. To aid the rapid development of vaccines for emergency use, parameters related to quality control and their specifications should, at the minimum, include the basic requirements of safety and effectiveness, while keeping in mind the urgency in rolling out vaccines for epidemic prevention and control. In view of the limited data generated during the R&D of quality control methods for new vaccines, some leeway should be permitted on the condition that specifications are continuously improved and validated in the subsequent R&D efforts.

In terms of quality control methods and specifications, China has set forth technical requirements on key items like viral strains, cells, purified bulk, and final bulks to final lots for vaccine production by each platform. These form part of the quality control effort and require registration of related products that are specifically used in the development of vaccines from any of the five technical platforms.

For a variety of cell lines involved in the production of COVID-19 vaccines, the relevant international guidelines were closely integrated with the Chinese pharmacopoeia, in order to formulate comprehensive recommendations on the quality control measures for the processes using cell lines in the production of vaccines. The numbers of new methods available for monitoring a range of parameters related to cell cultures for guaranteeing the quality have surged recently. For instance, a variety of methods for detecting contamination and misidentification of cell lines like multiplex polymerase chain reaction (PCR), DNA barcoding, and STR mapping are now available for rapidly identifying problems and quality issues with cell lines. In particular, the newly established methods for the rapid detection of exogenous contamination (such as the Touchdown PCR detection method for mycoplasma) not only guarantee the safety of the vaccine, but also solve the problem of lack of cell detection technology, which can save considerable time for vaccine R&D, while providing strong support for the quality of production processes during technical reviews.

**Fig. 1:**
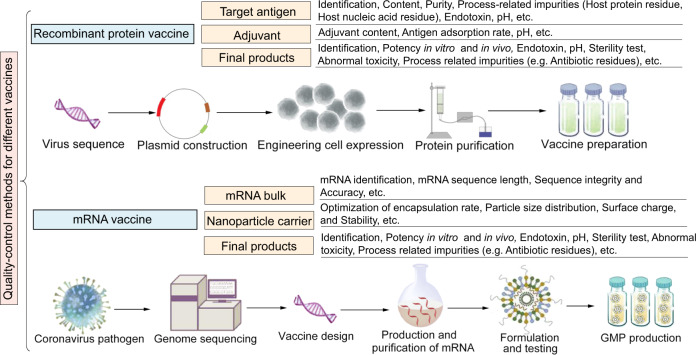
Key points of quality control for COVID-19 vaccines developed by different platforms. Note: the main quality control items are listed in the figure

For vaccines made up of recombinant protein (Fig. 1), the target antigen and adjuvant are considered as key quality attributes. Specifications on quality control for a list of items, including bulks and finished product, are put forward to R&D enterprises. For example, quality requirements for bulks focus on meeting the standards on the purity of the target antigen (active ingredient), content, residual impurities, etc. Generally, the requirements for passing quality control are similar to those prescribed for any existing vaccine that is similar to the candidate. The basic standards are adhered to even under compelling situations. Items that are difficult to be verified in a short period of time, such as protein N-terminal sequencing, are categorized as research items. At the initial stage, the results of research items are provided voluntarily and are not mandatory for meeting the quality control requirements for the time being. They are likely to be included as a quality inspection procedure at a later stage after validation of the empirical methodology along with process scale-up. In terms of finished products, depending on the performance of the product on the main verification items, data on at least two items pertaining to the testing of the efficacy in vivo and in vitro are required to ensure the consistency of the product quality.

For vaccines based on mRNA technology (Fig. 1), there is not much data to fall back on since they have been developed and deployed for human vaccination for the very first time, and different from other vaccines. In these types of vaccines, target mRNA is introduced inside the cells, the gene is translated and the protein is displayed on the surface of the cell. The key to the success is transgression of the plasma membrane of host cell by the target mRNA, which is achieved by selecting an appropriate cationic polymer in the vaccine formulation or lipid-based nanoparticles that can encapsulate the target mRNA. Since most of the technology involved has not been tested in real world situations, the technical guidelines for pharmaceutical research of COVID-19 preventive mRNA vaccine should be formulated timely and promulgated to guide the R&D. In guideline promulgated in China, in addition to the quality of the mRNA bulk, the quality of the nanoparticle carrier is also crucial for the effectiveness and safety of the vaccine, and therefore should be optimized diligently.^37^ The quality control of the mRNA bulk targets steps like mRNA identification, mRNA sequence length, sequence integrity and accuracy, etc. Research for enhancing the quality of nanoparticles should focus on optimization of encapsulation rate, particle size distribution, surface charge, stability and immunogenicity, etc. Biological activity of the final products should be demonstrated in vitro, as well as in vivo tests.

In addition, in the general guideline for RNA vaccines newly formulated by WHO in 2020,^46^ it also puts forward relevant requirements for vaccine design. For example, the rationale for the selection of the target antigen(s), encoded proteins (e.g., cytokines) and the inflammatory nature of the given mRNA, the quality, quantity and bias of the immune responses (e.g., T helper (Th1/2) cell phenotype), biostability, etc. should be clarified.

### Research on vaccine quality control methods

One of the key challenges in COVID-19 vaccine development that remains unaddressed is the drafting and acceptance of criterion for the evaluation of the efficacy of vaccines by all stake holders. In the early stages of COVID-19 vaccine development, NIFDC of China successfully prepared and established the SARS-CoV-2 neutralizing antibody (NtAb) standard, created the pseudovirus detection method as a substitute for tests involving use of live SARS-CoV-2 and ACE2 transgenic mice models for in vivo testing of the efficacy and safety of the vaccine. It is mandatory for the emergency use vaccines to provide data related to the use of these standards and methods in the preclinical studies before entering the clinical trials, leading to their subsequent approval for emergency use.

#### Preparation of SARS-CoV-2 antiserum

SARS-CoV-2 recombinant RBD protein was used to immunize goats and rabbits to successfully prepare several anti-goat and anti-rabbit sera against the virus. The highly potent sera were distributed to several Chinese manufacturers of SARS-CoV-2 inactivated vaccine, which solved the technical bottleneck of identification of the virus specifically and detection of exogenous virus-related factors.^47^ In addition, the preparation of serum has also helped some vaccine development companies to establish a vaccine antigen detection method.

#### Testing efficacy of candidate vaccines in vivo

For the first generation of coronavirus vaccines being developed and approved for emergency use, in vivo efficacy testing remains the main rate-limiting step for licensure. The traditional methods for establishing the efficacy of the vaccine rely on the collection of blood samples for the detection of antibodies 1 month after immunization. Based on the results from previous research, it is proposed that both the inactivated and recombinant protein vaccines should be tested for binding antibodies 14 days after the administration of the first dose of vaccine. After successful demonstration of the required titer of antibodies, the candidate vaccine for emergency use can successfully complete the registration requirements in the first attempt and quickly enter the clinical trial.

#### Establishing a universal method for detection of antigens in vaccine

Many R&D enterprises that developed inactivated vaccine and recombinant protein vaccine adopted their own antigen detection methods, which make it difficult to compare the active content of vaccines across the manufacturers. Recently, a team of scientists extensively screened monoclonal antibody libraries to obtain antibodies suitable for establishing a double antibody sandwich ELISA-based kit for the universal detection of antigens, which showed good correlates between vaccines developed from different platforms and different systems (unpublished date). Such a kit would be used for the evaluation of the in vitro efficacy of recombinant protein vaccines and inactivated vaccines.

### Research on preclinical and clinical evaluation methods

#### Establishment of a neutralizing antibody detection method based on pseudovirus

Elicitation of neutralizing antibodies is one of the main measures of vaccine pharmacodynamics during clinical trials. Plaques reduction and microcytopathic methods are the gold standards for the detection of NtAb at present. But, the application and popularization of these methods are greatly limited due to the need of live virus. In the early stage of the outbreak of COVID-19, Wang et al. constructed the SARS-CoV-2 pseudovirus using the VSV pseudovirus system and established the pseudovirus neutralization method. This method of detection of neutralizing antibodies offers several advantages like objectivity, sensitivity, and accuracy. Most importantly, there is no need for a P3 laboratory for performing tests using this method. Consequently, it has been used in many vaccine R&D enterprises and institutions, solving the key issue of detection of NtAb evoked by COVID-19 vaccine, and providing strong technical support for ensuring effectiveness of the process of evaluation for the vaccine.^48^

#### Establishment of transgenic animal models

Animal models are key tools for evaluating the amount of protection conferred by vaccines in preclinical studies. Using CRISPR/Cas9 knock-in technique, Sun et al. constructed a transgene mouse model that could express human ACE2 (HACE2) receptor. Compared with wild-type C57BL/6 mice, both young and old HCE2 mice maintained higher viral loads in the lungs, trachea, and brain during nasal infection. At the same time, increased interstitial pneumonia and cytokines were observed in SARS-CoV-2-infected HCE2 mice. At present, this model is being used in the preclinical pharmacological evaluation of several vaccines, providing a crucial tool for evaluating the ability of vaccine to confer protection against viral challenge.^49^

## Development and application of standard materials

Standard materials are yardsticks for ensuring the safety, effectiveness, and produce consistency of the vaccines. The use of rational, reliable, stable, and uniform standard materials is necessary to ascertain that the internal, as well as external regulatory QC requirements are met. These attributes also play a role in the evaluation of vaccines of different kinds originating from different regions or institutes involving various methods used for their appraisal. Such as enterovirus 71 (EV71) vaccines, methods for QC and establishment of standard reference materials for EV71 vaccines were done in the early stage of development to guarantee accurate evaluation of vaccines.^50,51^ Synchronous R&D of the vaccine by different manufacturers helped establish, authenticate, and validate the processes and methods for QC during preclinical and clinical immunogenic evaluation of vaccines produced by different manufacturers,^52–60^ which ensured the accuracy and comparability of vaccine, and aided the marketing and application of EV71 vaccines.^61^

Compared to EV71 vaccine, which was developed using only one technical platform, the establishment and application of standard materials for COVID-19 vaccines that have been developed from multiple platforms would be more challenging. Given the importance and need for unifying protocols for ease of comparison of different COVID-19 vaccines, the guiding principles on establishment and use of standards could greatly reduce the burden and challenges, surrounding approvals for these new vaccines.

### Establishment of national standard for neutralizing antibody

NtAb response is a key measure of immunogenicity and efficacy of vaccine. To assist in the evaluation of the efficacy of COVID-19 vaccines during the R&D stage, Hou et al. employed convalescent sera of COVID-19 to develop a national standard for SARS-CoV-2 NtAb with an assigned unitage of 1000 U/ml.^62^ This standardized reference also plays an important role in assuring adequate QC measures for vaccines developed from different platforms and by different manufacturers. Importantly, it provides a reference point for comparison of NtAb titers evoked by different vaccines. WHO established the first WHO international standard for anti-SARS-CoV-2 immunoglobulin (human; 20/136) in December 2020, and it was assigned an international unitage of 250 IU/ampoule. The standard was a result of an international collaborative effort with laboratories from all over the world participating in the calibration of the standard, leading to a uniform international standard for the estimation of antibody titers.^63^

### Establishment of uniform antigen reference materials for SARS-CoV-2

RBD is a universally accepted active domain of SARS-CoV-2 vaccine.^64^ Therefore, RBD protein content is the key of the whole virus inactivated vaccine and other forms of recombinant protein vaccines developed using various expression systems. Presence of correctly folded RBD ensures efficacy. To establish a uniform reference to quantify effective components in vaccine, Liang et al. selected a CHO-expressed RBD antigen that had a moderate antigen activity from different candidates. This RBD protein was distributed to 11 labs for collaborative calibration. The results indicated that this candidate standard showed good parallelism and linearity with inactivated antigen and S or RBD antigens produced from CHO cells, yeast, *Escherichia coli*, or sf9 cells, which demonstrated that this candidate standard could be used as a uniform antigen standard for inactivated or recombinant vaccines. The standard is expected to provide a uniform benchmark for the quantification of effective components in vaccine.

## Issues and prospects

The outbreak of COVID-19 has been catastrophic for human health, and its epidemiology posed a great challenge for global regulators and vaccine industry. In order to promote the R&D of vaccines and ensure the safety and effectiveness of vaccines, global regulators have formulated a series of guiding principles for the R&D of this new vaccine, including quality control, evaluation, and licensure. Among them, China is facing the biggest challenge due to the complicated and diverse vaccine R&D situation. In China, as a part of the national supervision system for vaccine development, CDE and NIFDC have played a leading role in launching the research on QC and evaluation at the earliest stages in collaboration with relevant laboratories involved in the vaccine R&D, which supported and accelerated the process of conditional marketing and emergency use of different vaccines with high quality.

In this process, the Chinese National Regulation Authority believes that the establishment of national and international unified evaluation methods and standards is extremely important for the QC and evaluation of these new vaccines. So, QC and evaluation methods, including psedovirus-based NtAb detection method, and NtAb standard, were rapidly developed, established and used in the quality control and evaluation of COVID-19 vaccines. Those accomplishments relied on the close communication and collaboration between Chinese regulatory authorities and international organizations (e.g., WHO), and the close cooperation among institutions with supervisory roles in China, and the direct communication among supervisors and manufacturers. These works unified projects, methods, and standards for QC of vaccines using same platforms to great extent, which ensures the consistency, comparability, and objectivity of quality control and evaluation of different kinds vaccines or same kinds vaccines developed by different manufactures.

Recently, COVID-19 inactivated and adenovirus-vectored vaccines have been approved for marketing. However, there has been variability in their protective efficacies, which needs to be further investigated. Furthermore, the recent variants documented in South Africa and Brazil might escape the protective immune responses elicited by these vaccines.^65,66^ More innovative technologies and ideas, such as research on heterologous prime-boost strategy of combining different vaccine candidates, next-generation vaccines capable of evoking more potent and effective immune responses against mutants, monovalent or multivalent vaccines, and the monitoring for tendency of the virus mutation should be studied if it can help mitigate these issues. At the same time, how to constantly adjust the standards and technical requirements of vaccines according to the changing situation, so as to ensure the safety and effectiveness of vaccines is a common challenge faced by global regulatory agencies. Active and close international cooperation and exchange is the only way to solve the problem to better prevent and control of the epidemiology of SARS-CoV-2 worldwide.
